# High Selectivity of 8-Hydroxyquinoline on *Leishmania (Leishmania)* and *Leishmania (Viannia)* Species Correlates with a Potent Therapeutic Activity In Vivo

**DOI:** 10.3390/ph16050707

**Published:** 2023-05-07

**Authors:** Sarah Kymberly Santos de Lima, Jéssica Adriana Jesus, Cristiano Raminelli, Márcia Dalastra Laurenti, Luiz Felipe Domingues Passero

**Affiliations:** 1 Institute of Biosciences, São Paulo State University (UNESP), Praça Infante Dom Henrique, s/n, São Vicente 11330-900, Brazil; sarahkslima@gmail.com (S.K.S.d.L.); jessica.dolly@hotmail.com (J.A.J.); 2 Laboratory of Pathology of Infectious Diseases (LIM50), Department of Pathology, Medical School of São Paulo University, São Paulo 01246-903, Brazil; mdlauren@usp.br; 3 Instituto de Ciências Ambientais, Químicas e Farmacêuticas, Universidade Federal de São Paulo, Diadema 09920-000, Brazil; raminelli@unifesp.br; 4 Institute for Advanced Studies of Ocean, São Paulo State University (UNESP), Rua João Francisco Bensdorp, 1178, São Vicente 11350-011, Brazil

**Keywords:** leishmaniasis, cutaneous leishmaniasis, treatment

## Abstract

Leishmaniasis is a neglected disease caused by protozoa of the genus *Leishmania*, which causes different clinical manifestations. Drugs currently used in the treatment such as pentavalent antimonial and amphotericin B cause severe side effects in patients, and parasite resistance has been reported. Thus, it is necessary and urgent to characterize new and effective alternative drugs to replace the current chemotherapy of leishmaniasis. In this regard, it has been experimentally demonstrated that quinoline derivatives present significative pharmacological and parasitic properties. Thus, the aim of this work was to demonstrate the leishmanicidal activity of 8-hydroxyquinoline (8-HQ) in vitro and in vivo. The leishmanicidal activity (in vitro) of 8-HQ was assayed on promastigote and intracellular amastigote forms of *L. (L.) amazonensis*, *L. (L.) infantum chagasi*, *L. (V.) guyanensis L. (V.) naiffi*, *L. (V.) lainsoni*, and *L. (V.) shawi*. Additionally, the levels of nitric oxide and hydrogen peroxide were analyzed. The therapeutic potential of 8-HQ was analyzed in BALB/c mice infected with a strain of *L. (L.) amazonensis* that causes anergic cutaneous diffuse leishmaniasis. In vitro data showed that at 24 and 72 h, 8-HQ eliminated promastigote and intracellular amastigote forms of all studied species and this effect may be potentialized by nitric oxide. Furthermore, 8-HQ was more selective than miltefosine. Infected animals treated with 8-HQ by the intralesional route dramatically reduced the number of tissue parasites in the skin, and it was associated with an increase in IFN-γ and decrease in IL-4, which correlated with a reduction in inflammatory reaction in the skin. These results strongly support the idea that 8-HQ is an alternative molecule that can be employed in the treatment of leishmaniasis, given its selectivity and multispectral action in parasites from the *Leishmania* genus.

## 1. Introduction

Leishmaniasis is a parasitic disease caused by a protozoa of the genus *Leishmania*, and at least 20 species can infect and cause the disease in humans, domestic, and wild animals [1]. During the life cycle of *Leishmania* sp., two different forms of the parasite can be characterized such as promastigotes that are flagellated forms, adapted to live extracellularly attached to the intestinal epithelium of sand fly vectors of the genera *Phlebotomus* or *Lutzomyia*, and amastigote forms, which are adapted to survive intracellularly in special compartments known as parasitophorus vacuoles that are assembled in macrophages and dendritic cells rapidly after infection. *Leishmania* parasites are transmitted to vertebrates during the blood meal of a female sand fly as promastigote forms, which are rapidly phagocytosed by macrophages; in the parasitophorous vacuoles, promastigotes differentiate into amastigote [2,3]. After some cycles of asexual reproduction, the excessive number of intracellular amastigotes lyses macrophages, and extracellular amastigotes infect nearby macrophages in the skin, or even reach other parts of the tegument as well as visceral organs such as the liver, spleen, or bone marrow [4,5]. However, other mechanisms of macrophage infection have been described; in this case, amastigote forms surrounded by the host cell membrane are transferred from an infected cell to another cell; this silent way of infection may be an important tactic to evade the immune response of the host [6].

Depending on the infecting species and localization of parasites in the body, different clinical forms of leishmaniasis can be diagnosed such as cutaneous or visceral leishmaniasis. In particular, cutaneous leishmaniasis (CL) exhibits a wide spectrum of clinical manifestations such as localized cutaneous leishmaniasis (LCL), which usually develops as an ulcerated lesion at the site of the sandfly bite, is painless and generally self-healing, but eventually leads to the development of a permanent scar, creating a stigma in patients [7,8]. Furthermore, LCL caused by *L. (L.) amazonensis* and *L. (V.) braziliensis* can progress to a more severe manifestation such as anergic cutaneous diffuse leishmaniasis (ACDL) and mucocutaneous leishmaniasis (ML), respectively [9].

Despite the undoubtful epidemiological and medical significance of *L. (L.) amazonensis* and *L. (V.) braziliensis,* other species also exhibit great medical and scientific importance in the South and Central Americas because they are ethological agents of CL such as *L. (V.) guyanensis, L. (V.) shawi, L. (L.) lainsoni, L. (V.) panamensis, L. (V.) naiffi*, and *L. (L.) infantum chagasi*, which was isolated from the skin of Honduran patients exhibiting atypical cutaneous leishmaniasis [10,11]. In general, parasites multiply and persist within tissue macrophages because they are able to suppress the development of a Th1 immune response, in particular, intracellular parasites suppress the production of IFN-γ and TNF-α cytokines, which activate macrophages to a leishmanicidal state. In contrast, the acquired immune response observed in patients with leishmaniasis is related to a Th2 immune response with a high production of IL-4, IL-10, or TGF-β, cytokines that are not protective to the vertebrate host. Therefore, downregulation of the Th1 immune response with a consequent upregulation of the Th2 immune response allows the parasite to multiply in tissues, and consequently, lesions spread along the cutaneous surface [12].

Although the importance of Th1 and Th2 immune responses in leishmaniasis has been widely discussed, no immunoprophylactic products such as vaccines have been produced to inhibit leishmanial infection and disease progression in humans [13,14]. Thus, conventional drugs such as pentavalent antimonial, amphotericin B, and miltefosine are the only approach to restrain the parasite and inhibit disease.

Pentavalent antimonials, originally developed as antiemetic drugs, have been used as first-line treatment for leishmaniasis since 1940 [15]. In the intracellular environment, pentavalent antimonials become active only after their reduction into trivalent antimonials, which can inhibit leishmanial trypanothione reductase (TR), an enzyme responsible for protecting trypanosomatids from oxidative damage [16,17,18]. In this way, pentavalent antimonials keep trypanothione in its oxidized state, making it impossible to scavenge reactive oxygen species (ROS), leading to the death of the parasite [19]. Treatment with pentavalent antimonial is performed by intravenous or intramuscular routes, however, even in systemic administration, this drug displays low selectivity in humans, causing several local and systemic side effects including abdominal cramps, nausea, weakness, cardiotoxicity, hepatotoxicity, and pancreatitis [20,21,22]. The emergence of parasites resistant to antimonials is a constant concern that impacts the cure rates, as observed in Bihar (India), where 60% of cases were refractory to antimonial treatment [23,24]. In Brazil, recent studies showed a variation of 60% to 90% in the cure rate, associated with 15% of cases that developed cardiotoxicity, hepatotoxicity, and pancreatitis during treatment [25].

Amphotericin B has been used as a second-line treatment for leishmaniasis, mainly in cases where antimonials are not effective due to the presence of resistant parasites or by low patient compliance to the treatment [26]. This drug is an antifungal antibiotic that binds to *Leishmania* ergosterol, leading to the formation of pores in the cell membrane, which allow the passage of ions, macromolecules, and water through the lipid bilayer; this hydric and ionic disbalance causes the death of *Leishmania* sp. Although a high rate of efficacy has been observed during the treatment of leishmaniasis, amphotericin B also binds to cholesterol, the main component of the mammalian cell membrane, causing severe side effects in humans that include fever, chills, arthralgia, nausea, vomiting, and headache, in addition to nephrotoxicity, which is observed in approximately 53% of patients [27]. Furthermore, this treatment is prolonged and requires medical monitoring, making it unsatisfactory to patients. On the other hand, amphotericin B entrapped into liposomes such as Ambisome^®^ exhibits low toxicity while maintaining efficacy in comparison to conventional amphotericin B [28,29]; this happens because liposomes are able to penetrate into the main organs affected by parasites, especially the spleen and liver, which in fact minimize the interaction of amphotericin B with cholesterol from the host cell membrane, and as a consequence, the toxicity is reduced in patients. However, the costs associated with this treatment limit the use of such formulations in low-income countries [27].

Miltefosine has been considered another second-line drug to treat leishmaniasis, and it is only available to be employed as an oral treatment. This drug is an alkylphosphocholine that was originally developed in 1940 as an anti-tumoral drug [30]. In *Leishmania*, miltefosine affects the phospholipid metabolism of the cell membrane, modifies the mitochondrial membrane potential, and induces programmed cell death [31], possibly by inhibiting cytochrome C oxidase [32]. Despite being initially considered a drug with high therapeutic activity, cases reported in India and Nepal showed an increased resistance to treatment, suggesting that the efficacy of treatment depends on the species of *Leishmania* involved in the infection [33]. However, in countries where *L. (L.) amazonensis* and *L. (L.) mexicana* are predominant, the cure rate for miltefosine is only 53%, significantly lower than the cure rate for antimonials [34]. Although oral treatment is attractive, miltefosine can also induce significant side effects including gastrointestinal, renal, and liver toxicity, besides teratogenicity in pregnant women [35].

Thus, it is possible to observe that none of the drugs employed in the therapy was developed specifically for leishmaniasis. Additionally, the efficacy of such drugs depends on the clinical form, infecting species, immunity, and health conditions of the patients; furthermore, the presence of resistant strains in nature may aggravate this scenario. Therefore, it is crucial and urgent to characterize new alternative drugs to be introduced in the therapy of leishmaniasis [36].

Quinoline is a heterocyclic molecule displaying a diversity of pharmacological activities such as antibacterial, anti-inflammatory, and antiparasitic properties [37]. The quinoline core allows the synthesis of different structures such as 8-hydroxyquinoline (8-HQ), which was first synthesized in 1953 [38], and so far, different works have shown that this molecule exhibits considerable pharmacological activities such as anticancer, antibacterial, antifungal, anti-inflammatory, and antiparasitic properties [39]. In leishmaniasis, a recent study demonstrated that 8-HQ as well as other quinoline derivatives were able to eliminate promastigote and amastigote forms of *L. (L.) amazonensis* with high selectivity [40]. Furthermore, 8-HQ was also active on promastigote and amastigote forms of *L. (L.) infantum* and *L. (V.) braziliensis* [41], suggesting that this molecule has multispectral activity on *Leishmania* parasites. In addition, in vivo studies have shown that the subcutaneous injection of 8-HQ in BALB/c mice infected with *L. (L.) amazonensis* reduced tissue parasitism [41,42,43]. Despite such elegant studies, to the best of our knowledge, the activity of 8-HQ has not been explored in other etiological agents of leishmaniasis or even administered as an intralesional drug. Once the multispectral property of such molecules and the therapeutic activity as an intralesional drug are proven, data may enlighten the importance of 8-HQ as an alternative antileishmanial agent.

Considering the urgency to characterize new drugs with antileishmanial activity, the present study demonstrated for the first time that 8-HQ is able to eliminate, with high selectivity, parasites that cause cutaneous leishmaniasis, and once administered by the intralesional route, is effective to decrease the lesion size and parasite load in experimental animals infected with a strain of *L. (L.) amazonensis*, which causes anergic diffuse leishmaniasis in humans. Furthermore, animals treated with 8-HQ upregulated the levels of the IFN-γ cytokine, which may support the elimination of parasites. Thus, this study demonstrates that 8-HQ is an interesting prototype drug to be used in leishmaniasis.

## 2. Results

### 2.1. In Vitro Studies

**Figure 1:**
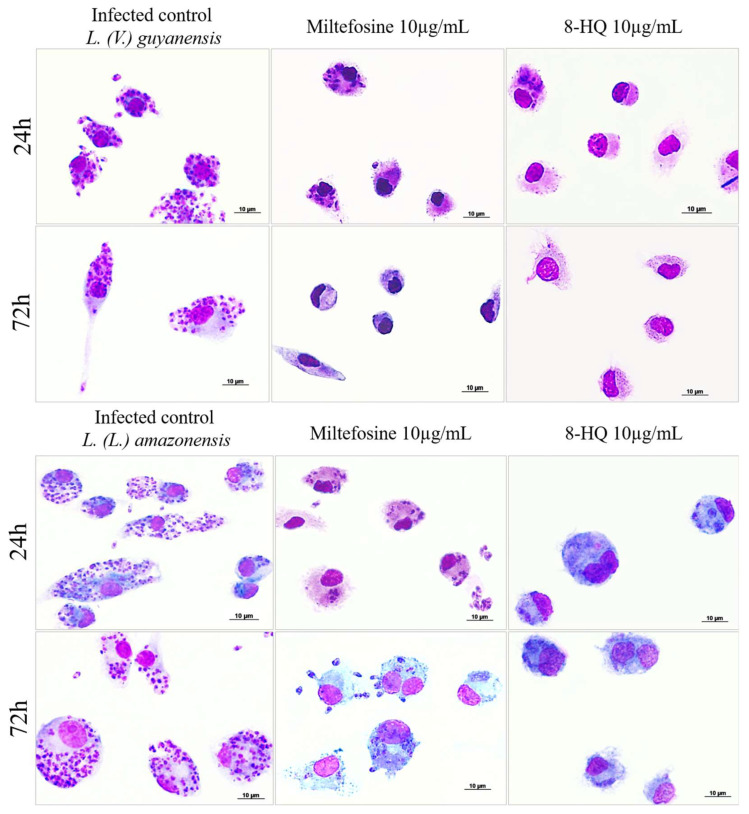
Bone marrow derived macrophages were infected with *L. (V.) guyanensis* or *L. (L.) amazonensis* for 24 h, followed by incubation with different concentrations of 8-HQ (1.25–10 μg/mL) or miltefosine (2.5–10 μg/mL) at 24 h or 72 h. The figures displayed here illustrate the morphology of macrophages and amastigote within macrophages treated with 10 µg/mL of 8-HQ or miltefosine.

In promastigote forms, it has been observed that at 24 h, *L. (V.) shawi* was the most susceptible to treatment with 8-HQ, displaying an effective concentration 50% (EC_50_) of 0.2 ± 0.03 μg/mL (Table 1), and at 72 h, *L. (V.) lainsoni* was the most affected, with an EC_50_ of 0.06 ± 0.01 μg/mL (Figure 1). Although 8-HQ also eliminated *L. (L.) amazonensis*, it was the most resistant amongst all of the assayed species at 24 and 72 h, showing an EC_50_s of 2.9 ± 0.3 and 1.1 ± 0.1 μg/mL, respectively (Table 1 and Figure 1). At 24 h and 72 h, 8-HQ eliminated promastigote forms of *L. (L.) infantum* with EC_50_ 2.1 ± 0.2 and 0.34 ± 0.1 μg/mL, *L. (V.) guyanensis* with 0.3 ± 0.08 and 0.1 ± 0.03 μg/mL, and *L. (V.) naiffi* with 0.8 ± 0.1 and 0.5 ± 0.08 μg/mL, respectively, and also *L. (V.) shawi* with an EC_50_ of 0.2 ± 0.03 and 0.31 ± 0.08 μg/mL (Table 1). Miltefosine, a standard drug, eliminated all parasite species at 24 and 72 h of incubation; promastigote forms of *L. (V.) shawi* were the most sensitive at 24 h of incubation, and *L. (V.) guyanensis* at 72 h, while *L. (V.) naiffi* were the most resistant at 24 h and *L. (L.) amazonensis* at 72 h (Table 1).

In macrophages, 8-HQ displayed a cytotoxic concentration 50% (CC_50_) of 36.3 ± 2.7 and 33.6 ± 2.2 μg/mL at 24 and 72 h of incubation, respectively. At the same time points, miltefosine eliminated macrophages with CC_50_ of 42.9 ± 1.3 and 32.8 ± 12.0 μg/mL, respectively.

In the amastigote forms, it was observed that at 24 h of incubation with 8-HQ, *L. (V.) lainsoni* and *L. (V.) shawi* were the most sensitive species (EC_50_ = 0.1 ± 0.09 and 0.1 ± 0.01 μg/mL) while *L. (L.) infantum* amastigote was the most resistant (EC_50_ = 2.0 ± 0.8 μg/mL); 8-HQ induced an intermediate leishmanicidal activity on *L. (L.) amazonensis* (EC_50_ = 1.9 ± 0.1 μg/mL), *L. (V.) guyanensis* (EC_50_ = 0.8 ± 0.1 μg/mL), and *L. (V.) naiffi* (EC_50_ = 0.45 ± 0.02 μg/mL). At 72 h of incubation, *L. (V.) naiffi* and *L. (V.) guyanensis* displayed high sensitivity to 8-HQ (EC_50_ = 0.03 ± 0.0002, and 0.03 ± 0.002 μg/mL, respectively), followed by and *L. (L.) infantum* and *L. (V.) shawi*; comparatively, *L. (L.) amazonensis* amastigote forms were the most resistant species to 8-HQ (Table 1). Intracellular amastigote forms of *L. (V.) lainsoni* treated with miltefosine were the most sensitive parasite and *L. (L.) amazonensis* was the most resistant at 24 h of incubation. At 72 h, intracellular amastigote forms of *L. (L.) infantum* were more sensitive and *L. (L.) amazonensis* was more resistant to miltefosine (Table 1).

Figure 1 illustrates the efficacy of 8-HQ and miltefosine at 10 µg/mL on intracellular amastigote forms of *L. (V.) lainsoni* (the most susceptible species to 8-HQ) and *L. (L.) amazonensis* (the most resistant species to 8-HQ) at 24 and 72 h.

The levels of hydrogen peroxide (H_2_O_2_) and intracellular nitric oxide (NO) were quantified in infected macrophages treated and non-treated with 8-HQ. In this regard, the levels of H_2_O_2_ were below the limit of detection, however, significant changes were observed in the intracellular NO.

**Figure 2:**
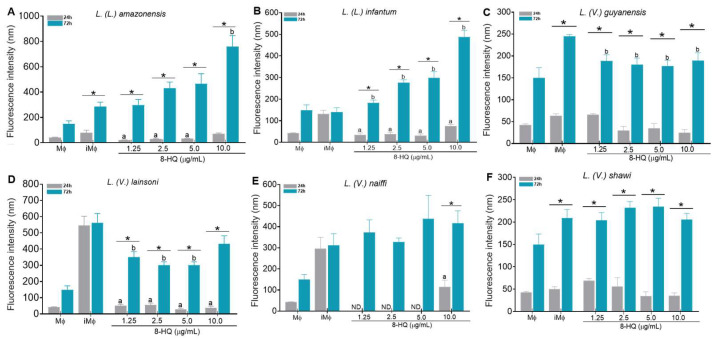
Intracellular nitric oxide production in macrophages (MΦ); infected macrophages (iMΦ); infected and treated macrophages. Macrophages were infected by *L. (L.) amazonensis* (**A**); *L. (L.) infantum* (**B**); *L. (V) guyanensis* (**C**); *L. (V.) lainsoni* (**D**); *L. (V.) naiffi* (**E**); *L (V) shawi* (**F**) treated with different concentrations of 8-HQ during 24 h or 72 h, and the level of NO was analyzed using the probe DAF-FM. * Represents significant differences (*p* < 0.05) between 24 h and 72 h. Significant difference (*p* < 0.05) in relation to iMΦ and the other groups is represented by the letter “a” at 24 h and the letter “b” at 72 h of incubation. All data were expressed through the mean and standard error. LPS was used as a positive control.

In macrophages infected with *L. (L.) amazonensis* (Figure 2A), *L. (L.) infantum* (Figure 2B), and treated with 8-HQ, it was observed that at 24 h of incubation, the treatment with 1.25–5 μg/mL reduced the levels of NO compared to the infected control (iMΦ) and control macrophages (MΦ), however, the infected macrophages treated with 10 μg/mL of 8-HQ restored the ability to produce NO in comparison to the controls. At 72 h of treatment, macrophages infected with *L. (L.) amazonensis* and treated with 10 μg/mL of 8-HQ was able to increase the levels of intracellular NO in comparison to the infected macrophages (*p* < 0.05), but in the infection caused by *L. (L.) infantum chagasi,* all concentrations of 8-HQ stimulated the production of NO in comparison to the infected control (*p* < 0.05). Comparatively, the levels of NO produced by macrophages were higher at 24 h than 72 h of incubation with 8-HQ (*p* < 0.05).

In the infection with *L. (V.) guyanensis* (Figure 2C), *L. (V.) lainsoni* (Figure 2D), *L. (V.) naiffi* (Figure 2E), and *L. (V.) shawi* (Figure 2F), a different pattern of NO production was observed. At 24 h of incubation, macrophages infected with *L. (V.) lainsoni* or *L. (V.) naiffi* that were submitted to incubation with all concentrations of 8-HQ showed a significant reduction in NO production compared to the respective infected controls. On the contrary, in infections caused by *L. (V.) guyanensis* or *L. (V.) shawi*, no modifications in NO were observed. At 72 h, macrophages infected with *L. (V.) lainsoni* and treated with 1.25–5 μg/mL exhibited a significant reduction in NO levels in comparison with the infected control, however, when infected cells were treated with 10 µg/mL, a restoration in the NO levels was observed compared to the levels of the iMΦ group. At 72 h, 8-HQ did not change the NO production in infections caused by *L. (V.) guyanensis*, *L. (V.) naiffi*, and *L. (V.) shawi* in comparison with the respective infected controls.

Furthermore, in treated cells, the levels of NO were higher at 72 h than at 24 h of incubation with 8-HQ. Macrophages incubated with LPS produced higher levels of NO during 24 (650 ± 53 fluorescence unities) or 72 h (820 ± 41 fluorescence unities) in comparison to all of the experimental conditions.

The production of NO from the control macrophages (MΦ) were considered the background of the reaction.

### 2.2. Efficacy of 8-HQ Administered by the Intralesional Route

In the present study, the efficacy of 8-HQ given by the intralesional route was assayed in the experimental cutaneous leishmaniasis caused by a strain of *L. (L.) amazonensis* that causes anergic diffuse cutaneous leishmaniasis in humans. In this case, the treatment of BALB/c was started in the sixth week post infection (PI). Glucantime was used as a standard treatment.

**Figure 3:**
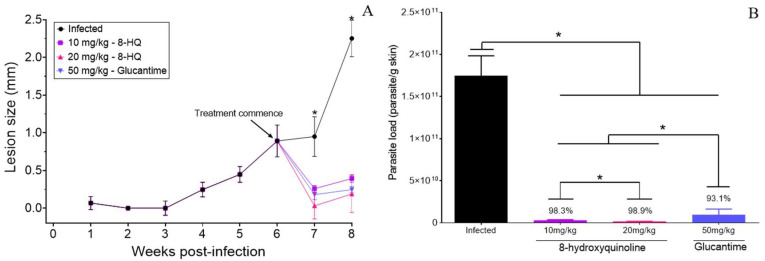
BALB/c mice were infected with 10^6^ promastigote forms of *L (L.) amazonensis* at the base of the tail; at the sixth week PI, intralesional treatment was started with 8-hydroxyquinoline (10 and 20 mg/kg) or glucantime (50 mg/kg). Animals were treated once a day for 10 days. Lesion development was monitored with a micrometer (**A**) and at the eighth week PI, parasite loads (**B**) were determined by the limiting dilution assay technique. * *p* < 0.05 between infected and treated groups at 0.05.

At 7- and 8-weeks PI, a significant decrease in the lesion size of animals treated with 10 and 20 mg/kg of 8-HQ was observed in comparison with the infected group (*p* < 0.05). Animals treated by the intralesional route with 50 mg/kg of glucantime also presented a significant decrease in the cutaneous lesions (Figure 3A). It was not possible to observe significant differences between animals treated with 8-HQ and glucantime because all treatments were able to decrease the cutaneous lesions to similar values (*p* > 0.05).

In comparison to the infected group, the skin parasite load decreased by 98.3, 98.9, and 93.1% in animals treated with 8-HQ (10 or 20 mg/kg) and glucantime (50 mg/kg) by the intralesional route, respectively (*p* < 0.05), as observed in Figure 3B. Comparatively, the treatment with 10 and 20 mg/kg of 8-HQ by the intralesional route was more effective at decreasing the number of amastigote forms than 50 mg/kg of glucantime (*p* < 0.05). Furthermore, animals treated with 20 mg/kg exhibited lower parasites than the group treated with 10 mg/kg of 8-HQ (*p* < 0.05), as demonstrated in Figure 3B.

### 2.3. Histopathological Changes

**Figure 4:**
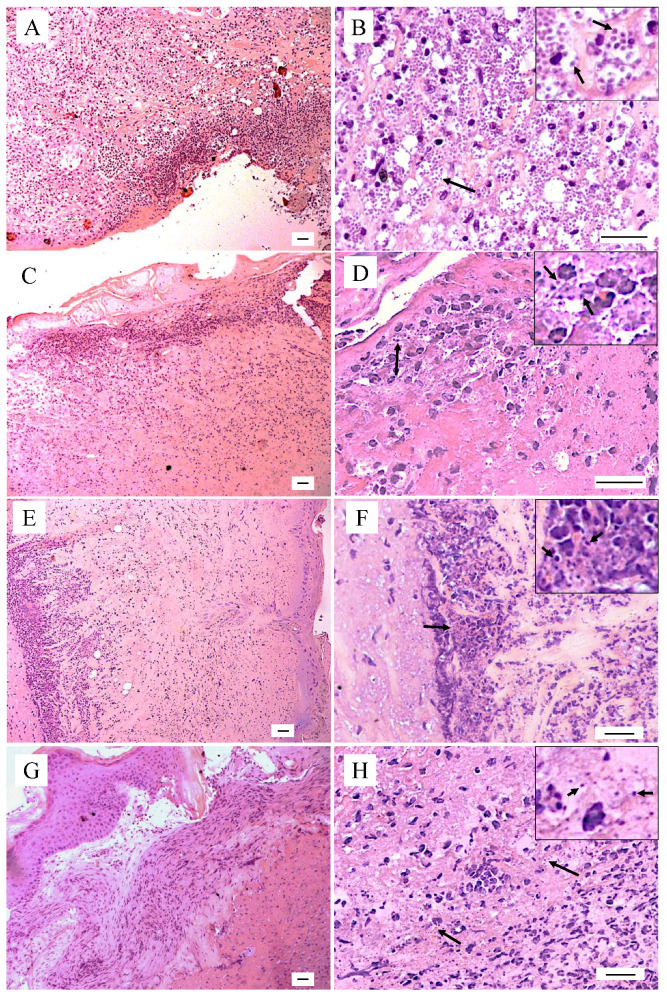
Histological skin sections of BALB/c mice infected with *L. (L.) amazonensis*. (**A**,**B**) Infected control (**C**,**D**) treated by the intralesional route with 10 mg/kg of 8-hydroxyquinoline; (**E**,**F**) treated by the intralesional route with 20 mg/kg of 8-hydroxyquinoline; (**G**,**H**) treated by the intralesional route with 50 mg/kg of glucantime. (**A**,**C**,**E**,**G**) show details of the epidermis and dermis of each group while figures (**B**,**D**,**F**,**G**) show the details mainly on the parasitism, illustrated by the black arrows (bars: 20 μm).

The infected control group showed an intense and diffuse inflammatory infiltrate spread through the dermis (Figure 4A), characterized by a large number of heavily infected macrophages (Figure 4B, detailed in inset in B), with few polymorphonuclear cells and lymphocytes (Figure 4B). Compared to the control, the animals treated by the intralesional route with 10 or 20 mg/kg of 8-HQ (Figure 4C, E, respectively) presented a mixed inflammatory infiltrate predominantly composed of lymphocytes with few polymorphonuclear cells and a reduced number of infected macrophages, demonstrating the therapeutic potential of this molecule. Furthermore, animals treated with 10 mg/kg of 8-HQ showed focal areas of a moderated inflammatory infiltrate (Figure 4C) with fewer morphologically recognizable amastigote forms than the control (Figure 4D, inset in D). In contrast, animals treated with 20 mg/kg exhibited an inflammatory infiltrated limited to the deep dermis (Figure 4E), with few suggestive amastigote forms (inset in Figure 4F). This histopathological feature suggests regression of the inflammatory process, which correlates with a very low tissue parasitism observed in the histological sections and in limiting dilution assays (Figure 3). Similarly, animals treated with 50 mg/kg of glucantime, administered by the intralesional route, exhibited focal areas of inflammation in the skin, which was characterized by the presence of mononuclear cells such as lymphocytes and macrophages, some of them infected with a low number of amastigote forms (Figure 4H, inset in H).

### 2.4. Immunological Studies

**Figure 5:**
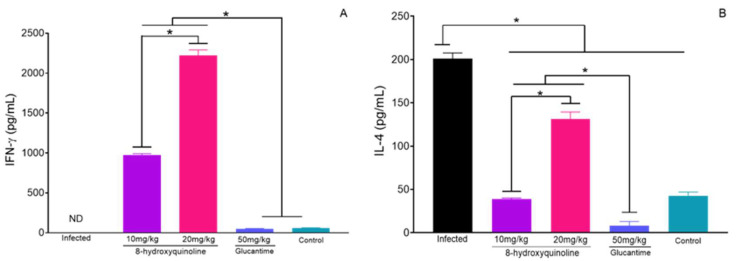
The draining lymph nodes of all of the experimental animals were collected, a single cell suspension was produced, adjusted to 5 × 10 cell/well, and cultured under stimulation with 5 μg of whole antigen of *L. (L.) amazonensis* for 72 h, when the supernatants were collected to quantify the amounts of IFN-γ (**A**) and IL-4 (**B**) by ELISA. * *p* < 0.05.

In the supernatant of mononuclear cells from infected animals, the IFN-γ cytokine was not detected (Figure 5A); in contrast, it was possible to detect high levels of IFN-γ in the supernatant of mononuclear cells from animals treated with 10 or 20 mg/kg of 8-HQ. Although the levels of IFN-γ in the supernatant of the mononuclear cells of animals treated with glucantime were upregulated compared to the infected group, the amount produced represents only a basal level of this cytokine, as observed in the control animals.

Mononuclear cells from the infected group produced high amounts of IL-4 cytokine (Figure 5B), however, mononuclear cells from animals treated with 8-HQ or glucantime produced reduced levels of this cytokine (*p* < 0.05). Comparatively, higher levels of IL-4 were detected in the supernatant of cells isolated from the lymph nodes of animals treated with 20 mg/kg of 8-HQ than in the cells from animals treated with 10 mg/kg of 8-HQ or glucantime, although it is important to highlight that mononuclear cells from animals treated with 20 mg/kg produced higher IFN-γ than IL-4, suggesting the development of a potent Th1 immune response.

Cells cultured with concanavalin A produced an elevated concentration of IFN-γ (3720 ± 189 pg/mL) and IL-4 (1089 ± 244 pg/mL), while cells cultured only with the medium did not produce detectable levels of such cytokines.

## 3. Discussion

Current treatments used in leishmaniasis cause severe side effects in patients, and in some areas around the world, the lack of adherence to medical treatment may lead to the emergence of parasites resistant to the main classes of drugs used in leishmaniasis. Thus, the characterization of new compounds and treatment schemes with a leishmanicidal effect on the majority of pathogenic species of *Leishmania* has become urgent. In this sense, 8-HQ is an interesting molecule to develop new and effective drugs for the treatment of neglected tropical diseases because its parasiticidal effect has already been demonstrated in infections caused by *Schistosoma mansoni* [44], *Toxoplasma gondii* [45], and *Leishmania* sp. [42,46,47,48]. Furthermore, the antileishmanial activity of 8-HQ alone or in combination has already been tested and the results published [49]. In the present manuscript, we expand the current knowledge on the activity of 8-HQ by showing the multispectral activity of this molecule on *Leishmania* sp. and demonstrate its efficacy in in vivo experiments by administering this alternative drug by the intralesional route to animals infected with a strain of *L. (L.) amazonensis* that causes anergic diffuse leishmaniasis in humans.

Experiments performed in vitro carried out with *L. (L.) amazonensis*, *L. (L.) infantum*, *L. (V.) guyanensis*, *L. (V.) lainsoni*, *L. (V.) naiffi*, and *L. (V.) shawi* showed that 8-HQ was able to eliminate promastigote and amastigote forms of all analyzed species at 24 and 72 h of incubation. In this regard, it was observed that promastigote and amastigote forms of the *Viannia* subgenus were the most susceptible species to 8-HQ, eliminating both forms of the protozoan with high selectivity. In addition, at 72 h of treatment, the compound was 18.36 and 11.02 times more selective for promastigotes of *L. (V.) lainsoni* and *L. (V.) guyanensis* than *L. (L.) amazonensis*, respectively, and 30 times more selective for amastigotes of *L. (V.) naiffi* and *L. (V.) guyanensis* than for *L. (L.) amazonensis*, the least susceptible species treated with 8-HQ. Similarly, *L. (V.) shawi* was the third most susceptible to the prototype drug in comparison to the other species evaluated, however, *L. (L.) infantum chagasi* was the second most resistant parasite to 8-HQ.

Although parasites from the subgenus *Leishmania* were less sensible to the action of 8-HQ than the *Viannia* parasites, they were still eliminated with high selectivity, mostly when compared to miltefosine, a standard drug used in leishmaniasis therapy. Previous studies carried out with promastigote forms demonstrated that at 48 h of incubation, 8-HQ eliminated *L. (L.) amazonensis* with an EC_50_ of 0.05 µg/mL (SI 328), and *L.(L.) infantum* with an EC_50_ of 0.26 µg/mL (SI 62) [41]. Similarly, *L. (V.) braziliensis* promastigotes were eliminated by the action of 8-HQ with an EC_50_ of 0.35 µg/mL (SI ~47). Furthermore, 8-HQ reduced intracellular amastigote forms of *L. (V.) braziliensis* with higher efficacy in comparison to amphotericin B [41]. Thus, the data presented herein corroborate the solid efficacy and selectivity of 8-HQ to eliminate *Leishmania* sp., and reinforce the inherent potential of such molecules to produce new and effective drugs to combat this infection. Furthermore, the SI, defined as the ratio between the cytotoxic concentration (CC_50_) and effective concentration 50% (EC_50_), estimated herein and in previous studies, indicated that 8-HQ has high leishmanicidal effect while inducing low cytotoxicity in macrophages. In the present study, at 24 h, the SI for 8-HQ in the in vitro infections ranged from 12.5 to 363; and at 72 h from 30.5 to 1120, while miltefosine at 24 h ranged from 1.8 to 85.8 and at 72 h from 2 to 656 These findings show the superior activity of 8-HQ over miltefosine.

Considering the high selectivity of 8-HQ toward *Leishmania* sp., it was investigated whether such activity is dependent on hydrogen peroxide and/or nitric oxide production. Some natural products increase the respiratory activity of host cells during phagocytosis and infectious processes, leading to the production of reactive oxygen and/or nitrogen species [50], which trigger a large spectrum of biological activities such as the elimination of intracellular pathogens [51]. In this regard, it was observed that 8-HQ did not stimulate the production of hydrogen peroxide in infected macrophages, but a curious behavior was observed with respect to NO production. In general, at 24 h of incubation with 8-HQ, a reduction or no changes in the NO levels in macrophages infected with *Leishmania* sp. was detected in comparison to the respective controls. It is possible that 8-HQ destroyed significant numbers of intracellular parasites, and the low number of amastigote forms was not enough to elicit NO production in such host cells, as observed in Figure 1. On the other hand, infected macrophages treated with 10 μg/mL of 8-HQ, which presented a very low number of amastigote forms, tended to normalize the levels of NO, suggesting that the high concentration of 8-HQ may present some modulatory activity at 24 h of incubation. In particular, such modulatory activity was potentialized at 72 h in infections caused by *Leishmania* subgenus. As observed in Table 1 and Figure 1, the species *L. (L.) amazonensis* and *L. (L.) infantum chagasi* were less susceptible to 8-HQ, which suggests that more intracellular parasites were viable than in infections caused by the *Viannia* subgenus, and as a consequence, macrophages already activated by 8-HQ produced more NO than the infected controls, which indeed suffered a massive intracellular infection.

In contrast, at 72 h of treatment, it was observed that infected macrophages with *L. (V.) guyanensis* and *L. (V.) lainsoni* treated with 8-HQ decreased the levels of NO, but no changes in NO levels were observed in macrophages infected with *L. (V.) naiffi* and *L. (V.) shawi*. Despite these events, all macrophages displayed a very low number of intracellular amastigotes, suggesting that the pro-inflammatory activity of such cells tends to be normalized. However, compared to the non-infected macrophages, such levels of NO should still be considered high, but it is possible that such effect might be related to the immunomodulatory activity induced by 8-HQ.

Previous studies have shown that *L. (L.) infantum-* and *L. (L.) amazonensis*-infected macrophages incubated with 8-HQ did not produce NO at 48 h of incubation [41,52]. The absence of intracellular NO in such studies should indicate that the production of NO is a late process in the treatment with 8-HQ, furthermore, it also suggests that immunomodulatory activity may play a secondary role in leishmanicidal mechanisms. However, BALB/c mice peritoneal macrophages infected with *L. (L.) amazonensis* and treated with an imidazoquinoline-based TLR7/8 agonist, a quinoline derivative, were able to eliminate amastigote forms of *L. (L.) amazonensis* associated with the production of NO and H_2_O_2_ after 72 h of incubation [53], suggesting that structurally-related molecules may share similar biological properties, and that NO stimulation is possibly a late process, as observed herein.

Considering that 8-HQ exhibited multispecies action that was more active and selective than miltefosine and was still able to modulate the microbicidal potential of macrophages, the therapeutic property of this molecule was assayed in the experimental model of cutaneous leishmaniasis caused by *L. (L.) amazonensis*. In this regard, BALB/c mice infected with *L. (L.) amazonensis* were treated with 10 mg/kg and 20 mg/kg of 8-HQ by the intralesional route; in addition, 50 mg/kg glucantime given via the intralesional route was used as the conventional drug used in human treatment. Both treatments were efficient at reducing the lesion size and tissue parasitism. However, the number of parasites in the skin was lower in animals treated with 10 and 20 mg/kg of 8-HQ in comparison with animals treated with the conventional treatment, suggesting that 8-HQ is an interesting prototype drug to be used in the treatment of leishmaniasis. In addition, 20 mg/kg of 8-HQ was more active at decreasing the parasite load in the skin than 10 mg/kg. Previous studies showed that BALB/c mice infected with *L. (L.) amazonensis* and treated with 8-HQ (10 mg/kg/day) was more active to eliminate tissue parasites than amphotericin B (5 mg/kg/day) [41,48].

In addition to the low parasitism observed in infected animals treated with 8-HQ, histopathology studies in the skin showed that this drug decreased the inflammatory infiltrate to focal areas that were characterized by a low number of macrophages with suggestive bodies, resembling amastigote forms. The histological pattern observed in the animals treated mainly with 20 mg/kg of 8-HQ suggests that the skin of the animals is in a healing process, in fact, the limiting-dilution assay allows us to conclude that very few parasites persisted in the skin, which may account for this. Although animals treated with glucantime exhibited a significative decrease in lesion size and low parasitism, in the histopathology analysis, amastigote forms could be easily recognized, which, in turn, had a correlation with a significant inflammatory response, composed of macrophages with a low number of amastigote forms. Corroborating our results, a previous study showed that synthetic quinoline alkaloids were active in hamsters infected with *L. (V.) panamensis* and in histopathological studies, it was observed that there was a significant reduction in the inflammatory infiltrate in comparison with the non-treated animals [54], suggesting that the high leishmanicidal activity induced by quinolines leads to a reduction in the inflammatory infiltrate.

In addition to a direct effect of 8-HQ on promastigote and amastigote forms, it was also observed that macrophages incubated with 8-HQ for 72 h exhibited an elevated production of NO in comparison with the controls. To analyze if this immunomodulatory property was kept in vivo, the production of the IFN-γ and IL-4 cytokines were quantified in the supernatant of mononuclear cells isolated from the lymph nodes of animals infected and treated with 8-HQ. These cytokines were strategically quantified in the murine model of leishmaniasis because the IL-4 cytokine induces the polarization of the immune response to a Th2 immune pole, in which macrophages become permissive to parasite multiplication. On the other hand, the IFN-γ cytokine induces the polarization of the immune response to a Th1 immune pole, causing the activation of infected macrophages to a leishmanicidal state by the action of NO [55,56]. In the present study, it was observed that mononuclear cells from animals treated with 10 and 20 mg/kg of 8-HQ produced high levels of IFN-γ in comparison to the infected animals, and it seems that the production of this cytokine was proportional to the concentration of the administered dose of 8-HQ. On the other hand, animals treated with glucantime produced only basal levels of IFN-γ.

In contrast to IFN-γ, it was seen that animals treated with 8-HQ exhibited a reduction in IL-4 levels compared to the infected control; despite a low concentration of IL-4 in the treatment, it is also important to observe that treatment with 20 mg/kg of 8-HQ increased the IL-4 levels, but even so, this group still produced higher amounts of IFN-γ than IL-4. Previous studies conducted with quinoline derivatives entrapped in micelle systems pointed to a high therapeutic activity associated with the development of a Th-1 immune response [47,57], which clearly potentialized the leishmanicidal activity of the treatment. These data reinforce that 8-HQ as well as other structurally related molecules can be used as an alternative molecule to treat cutaneous leishmaniasis.

## 4. Materials and Methods

### 4.1. Culture Media and Drugs

Schneider medium (Sigma-Aldrich, St. Louis, MO, USA) was supplemented with 10% of heat-inactivated fetal bovine serum (FBS), 10 μg/mL of gentamicin, and 1000 U/mL of penicillin (S10). RPMI 1640 medium (Thermo Scientific, Waltham, MA, USA) was supplemented with 10% inactivated fetal bovine serum (FBS), 1% 100× pyruvate, 1% of MEM solution of non-essential amino acids, 10 μg/mL of gentamicin, 1000  U/mL penicillin, and 0.1% of 2-mercaptoethanol (R10). Miltefosine and 8-hydroxyquinoline (purity >99%) were obtained from Sigma-Aldrich (St. Louis, MO, USA). Glucantime was obtained from Sanofi-Aventis (São Paulo, Brazil).

### 4.2. Animals

Six- to eight-week-old male BALB/c mice were obtained from the animal facility center of the Medical School of São Paulo University. This study was performed in accordance with the recommendations in the Guide for the Care and Use of Laboratory Animals of the Brazilian National Council of Animal Experimentation. The protocol was approved by the Committee on the Ethics of Animal Experiments of the Institutional Animal Care and Use Committee at the Medical School of São Paulo University (CEUA1648/2022). For all experimental procedures, the animals were anaesthetized with ketamine (100 mg/kg) and xylazine (10 mg/kg).

### 4.3. Parasite Strains

The parasites *L. (L.) amazonensis* (MHOM/BR/73/M2269), *L. (L.) infantum chagasi* (MHOM/HND/2017/AMA-65), *L. (V.) guyanensis* (MHOM/BR/1775/M4147), *L. (V.) lainsoni* (MHOM/BR/1981/M6426), *L. (V.) naiffi* (MHOM/BR/1981/M6426), and *L. (V.) shawi* (MCEB/BR1984/M8408) were identified using monoclonal antibodies and isoenzyme electrophoretic profiles at the Leishmaniasis Laboratory of the Evandro Chagas Institute (Belém, Pará State, Brazil). Parasite species were grown in S10 medium at 25 °C. In all experiments, parasites were in a stationary phase of growth, and they were in the first passage of culture to perform in vivo experiments.

### 4.4. Promastigote Assay

Promastigote forms of *L. (L.) amazonensis*, *L. (L.) infantum chagasi*, *L. (V.) guyanensis*, *L. (V.) lainsoni*, *L. (V.) naiffi*, and *L. (V.) shawi* in the stationary phase of growth were collected from the medium by centrifugation (1200× *g*, 10 min, 4 °C), and the parasite concentration was adjusted to 2 × 10^6^ parasite/well in a 96-well culture plate at 25 °C in S10 medium. Parasites were incubated with different concentrations of 8-HQ or miltefosine (0.08 to 100 μg/mL). Control parasites were incubated only with S10. At 24 and 72 h of incubation, the parasites were centrifuged at 1200× *g*, 10 min, 4 °C, and the supernatants were discarded. Parasites were washed three times with 200 μL of PBS and the viability was analyzed using PrestoBlue reagent (Thermo Scientific, Waltham, MA, USA). After being added to the cells, PrestoBlue (resazurin-based reagent) passively accesses the intracellular environment of *Leishmania* parasites and is converted in the intracellular environment by the redox machinery of viable cells into resorufin, a highly fluorescent compound. Therefore, the intensity of intracellular fluorescence is correlated with the number of viable parasites. After 30 min of incubation, plates were read in an ELISA reader at 416 nm excitation and 574 nm emission. For each parasite species, the drug concentration that eliminated 50% of parasites—effective concentration 50% (EC_50_)—was estimated using simple correlation analysis in GraphPad Prism 5.0 software [58].

### 4.5. Bone Marrow-Derived Macrophages (BMMs)

Bone marrow was flushed from the femurs and tibias of 7-week-old BALB/c mice using Hanks’ balanced salt solution (HBSS). After 2 wash steps at 2000 RPM, 5 min, 4 °C, red blood cells were lysed at 4 °C with 0.17 M NH_4_Cl, pH 7.4, for 5 min. The reaction was stopped by the addition of HBSS. Cells were centrifuged at 2000 RPM, 5 min, 4 °C, the supernatant was discharged, and cells were plated in a tissue culture-treated Petri dish with RPMI 1640 supplemented with 15% (vol/vol) of L929 cell-conditioned medium (LCM) as a source of colony-stimulating factor-1 (CSF-1) in a 37 °C incubator with 5% CO_2_. Twenty-four hours later, non-adherent cells were collected and cultured in sterile Petri dishes with R10 plus 15% of LCM for a week; at days 2, 3, 5, and 7, 15% of LCM was added to the culture. On day 8, adherent cells were collected with a cell scraper, counted, and plated in sterile 96-well plates or in round cover slips in 24-well plates.

#### 4.5.1. Cytotoxicity Assay

The BMMs were adjusted to 10^6^ macrophages/well in a 96-well plate; 24 h later, serial dilutions of 8-HQ or miltefosine (0.08 to 100 μg/mL) were added to the cells that were cultivated in a humidified incubator at 37 °C and 5% CO_2_. Cell cytotoxicity was analyzed at 24 and 72 h later. Control cells were incubated with R10 only. At each time point, cells were centrifuged at 1200× *g*, 10 min, 4 °C, and the supernatants were discarded. Macrophages were washed three times with 200 μL of PBS. Then, the cell viability was assessed using PrestoBlue according to Section 4.4. In summary, resazurin passively accesses the intracellular environment of macrophages; if they are viable, resazurin will be converted intracellularly into resorufin, a highly fluorescent compound. Thus, the intensity of intracellular fluorescence is correlated with the number of viable cells. Drug concentration that eliminated 50% of cell population—cytotoxic concentration 50% (CC_50_)—was estimated using simple correlation analysis in GraphPad Prism 5.0 software. The selectivity index (SI) of 8-HQ was estimated by the ratio between CC_50_ and EC_50_ [59].

#### 4.5.2. Intracellular Infection and Treatment (In Vitro)

BMMs were cultured in round coverslips (10^5^ macrophage/coverslip) in 24-well plates for 24 h, followed by infection with *L. (L.) amazonensis*, *L. (L.) infantum*, *L. (V.) guyanensis*, *L. (V.) lainsoni*, *L. (V.) naiffi*, and *L. (V.) shawi* promastigote forms at a ratio of 10 parasites per 1 macrophage in a humidified incubator with 5% CO_2_ at 35 °C. Twenty-four hours later, each well was washed three times with warm PBS to withdraw the free parasites. Then, 8-HQ or miltefosine (1.25–10 μg/mL) was added to the infected cells during 24 and 72 h. Infected controls were cultured only with R10. After the incubation periods, wells were washed three times with warm PBS, coverslips were dried at room temperature, fixed with methanol, and stained by Giemsa (Sigma-Aldrich, St Louis, MO, USA). At least 100 cells/coverslip were quantified and the infection indices were estimated according to the expression:

II = % Infected macrophages × Internalized amastigotes/Infected Macrophages

### 4.6. Determination of Intracellular Nitric Oxide Production

BMMs were plated in a 96-well black plate in R10 and placed in a humidified incubator with 5% CO_2_, 35 °C for 24 h. After this period, macrophages were infected with *L. (L.) amazonensis*, *L. (L.) infantum*, *L. (V.) guyanensis*, *L. (V.) lainsoni*, *L. (V.) naiffi* and *L. (V.) shawi* promastigote forms at a ratio of 10 parasites per 1 macrophage for 24 h. Twenty-four hours later, the wells were washed three times with warm PBS to remove free parasites and cells were incubated with 1.25 to 10 μg/mL of 8-HQ; controls were incubated with R10 only. Positive controls were incubated with 100 ng/mL of LPS (Sigma-Aldrich, St Louis, MO, USA) during 24 and 72 h. At these time points, cells were washed three times with 200 μL of warm PBS, followed by incubation with 5 mM of 4-amino-5-methylamino-2’,7’-difluorofluorescein diacetate (DAF-FM), which is a probe able to detect intracellular nitric oxide (NO). It reacts with NO and forms a highly fluorescent molecule, benzotriazole, which is detected by fluorescence readers. Cells were incubated for 60 min with DAF-FM, followed by three washes with warm PBS. Plates were read with 515 nm emission and 495 nm excitation. Blank controls, built with PBS, R10, and R10 plus drugs or LPS did not interfere with the fluorescence spectra of the reaction.

### 4.7. Infection and Treatment

Twenty male BALB/c mice were subcutaneously infected in the base of the tail with 10^6^ promastigote forms of *L. (L.) amazonensis* in the stationary phase of growth. Five animals received only sodium chloride 0.9% (*w*/*v*) under the same route (healthy group). Four weeks after infection, *L. (L.) amazonensis*-infected BALB/c mice were divided into five groups: group 1 (G1) and group 2 (G2) were constituted by infected animals injected by the intralesional route with 10 or 20 mg/kg of 8-HQ, respectively; group 3 (G3) was injected with 100 mg/kg of glucantime by the intralesional route; group 4 (G4) was the infected control, injected with the vehicle solution by the intralesional route; group 5 (G5) was constituted by non-infected, non-treated animals. The treatment was started in the sixth week post-infection, and the animals were treated for 10 consecutive days, once daily. The physical conditions of the animals were monitored once a week. One week after the last dose, the animals were euthanized with thiopental. Skin fragments were collected, fixed in formalin, and stained with hematoxylin and eosin to analyze the histological changes. There was no dead prior to the endpoint.

Of note, the species *L. (L.) amazonensis* was selected to analyze the efficacy of 8-HQ in vivo because it was the most resistant to the action of 8-HQ and importantly, this parasite reproduces a debilitating and progressive disease in BALB/c mice, that in fact mimics the most severe clinical forms of cutaneous leishmaniasis in humans. Therefore, this model is significant for experiments involving chemotherapy.

#### Clinical Course of Lesion Development and Determination of Parasite Burden in the Skin of Infected and Treated Animals

The clinical course of lesion development was evaluated once a week by recording the average diameter of the skin lesions using a caliper.

The parasite load in the skin was determined using the quantitative limiting dilution assay as previously described [60]. Briefly, a skin fragment from the base of the tail was aseptically excised, weighted, and homogenized in S10. The skin suspensions were diluted 1000 times and subjected to 12 serial dilutions (1:4) with four replicate wells. The number of viable parasites was determined based on the highest dilution that the promastigotes could be grown after 10 days of incubation at 25 °C in S10.

### 4.8. Quantification of Cytokines

The subiliac and popliteal lymph nodes from different groups were aseptically collected, macerated in the R10 medium, and the number of cells was estimated under Trypan blue exclusion dye. Mononuclear cells were adjusted at 5 × 10^5^ cells/well and stimulated with 5.0 μg of whole antigen of *L. (L.) amazonensis* or 1.0 μg of concanavalin A (positive control) for 72 h; negative controls were cultivated only with the R10 medium. Cells were cultured in a humidified incubator, 37 °C, 5% CO_2_. Following this experimental time, the supernatants were collected, and the amounts of IL-4 and IFN-γ (BD, Franklin Lakes, NJ, USA) in the supernatant of the cells were quantified by the sandwich enzyme-linked immunosorbent assay (ELISA) in accordance with the manufacturer’s recommendations.

### 4.9. Statistical Analysis

Values were expressed as the mean ± standard error. Statistical analyses were performed using GraphPad Prism 5.0 software, and the ANOVA test was used to analyze the differences between groups. Statistical significance was set at a *p* < 0.05.

## 5. Conclusions

In conclusion, the results presented herein reinforce that 8-HQ exhibits significant antileishmanial activity in vitro and high selectivity for all of the species studied. It is important to emphasize that the *Leishmania (Viannia)* species were more susceptible to the compound. Although species belonging to the subgenus *Leishmania* were relatively more resistant, they were successfully eliminated by the action of 8-HQ, and this effect was probably potentialized by the production of intracellular NO. Furthermore, the in vitro data reinforce that 8-HQ is more selective than miltefosine, an approved drug for leishmaniasis treatment. Finally, the in vivo data confirm that 8-HQ inhibited the progression of cutaneous disease in BALB/c and polarized the immune response toward a Th1 immune pole that was also associated with a reduction in the inflammatory reaction in the dermis of infected animals. Thus, the data presented herein suggest that 8-HQ is an interesting drug with high potential to be employed in the treatment of cutaneous leishmaniasis.

## Figures and Tables

**Table 1:** Promastigote and amastigote forms of *L. (L.) amazonensis, L. (L.) infantum, L. (V.) guyanensis, L. (V.) lainsoni, L. (V.) naiffi,* and *L. (V.) shawi* as well as bone-marrow differentiated macrophages were incubated with 8-HQ or miltefosine (a standard drug used in the treatment of leishmaniasis) for 24 or 72 h. Cytotoxic concentration 50 (CC_50_) on macrophages, effective concentration 50 (EC_50_) on promastigotes, and intracellular amastigotes were estimated. Selectivity indexes (SI) were calculated using the ratio between CC_50_ to EC_50_. Results were expressed as the mean ± standard error of three independent experiments. 8-HQ—8 hydroxyquinoline; Milt—miltefosine.

		EC_50_ (μg/mL)				CC_50_ (μg/mL)	
		(Selective Index—SI)					
		Promastigote Forms		Amastigote Forms		Macrophages	
		24 h	72 h	24 h	72 h	24 h	72 h
*L. (L.) amazonensis*	8-HQ	2.9 ± 0.3	1.1 ± 0.1	1.9 ± 0.1	0.9 ± 0.09		
		**SI (12.5)**	**SI (30.5)**	**SI (19.1)**	**SI (37.3)**		
	Milt	8.5 ± 0.4	16.1 ± 0.1	6.5 ± 0.06	3.5 ± 0.2		
		**SI (5.0)**	**SI (2.0)**	**SI (6.6)**	**SI (9.3)**		
*L. (L.) infantum chagasi*	8-HQ	2.1 ± 0.2	0.34 ± 0.1	2.0 ± 0.8	0.05 ± 0.001		
		**SI (17.3)**	**SI (98.8)**	**SI (18.1)**	**SI (672)**		
	Milt	19.3 ± 1.6	7.5 ± 1.5	4.1 ± 0.2	0.05 ± 0.01		
		**SI (2.2)**	**SI (4.4)**	**SI (10.4)**	**SI (656)**		
*L. (V.) guyanensis*	8-HQ	0.3 ± 0.08	0.1 ± 0.03	0.8 ± 0.1	0.03 ± 0.002		
		**SI (121)**	**SI (336)**	**SI (45.3)**	**SI (1120)**		
	Milt	5.0 ± 1.3	1.8 ± 0.63	4.9 ± 1.4	0.8 ± 0.2		
		**SI (8.6)**	**SI (18.2)**	**SI (8.8)**	**SI (41)**		
*L. (V.) lainsoni*	8-HQ	0.6 ± 0.1	0.06 ± 0.01	0.1 ± 0.09	0.5 ± 0.01		
		**SI (60.5)**	**SI (560)**	**SI (363)**	**SI (67.2)**		
	Milt	5.3 ± 0.1	2.4 ± 0.2	0.5 ± 0.03	1.8 ± 1.04		
		**SI (8.1)**	**SI (13.7)**	**SI (85.8)**	**SI (18.2)**		
*L. (V.) naiffi*	8-HQ	0.8 ± 0.1	0.5 ± 0.08	0.45 ± 0.02	0.03 ± 0.0002		
		**SI (45.4)**	**SI (67.2)**	**SI (80.6)**	**SI (1120)**		
	Milt	23.5 ± 5.7	13.7 ± 0.5	2.0 ± 0.3	0.1 ± 0.08		
		**SI (1.8)**	**SI (2.4)**	**SI (21.4)**	**SI (328)**		
*L. (V.) shawi*	8-HQ	0.2 ± 0.03	0.31 ± 0.08	0.1 ± 0.01	0.2 ± 0.001		
		**SI (181)**	**SI (108.4)**	**SI (363)**	**SI (168)**		
	Milt	1.7 ± 0.09	2.4 ± 0.69	3.9 ± 0.4	1.6 ± 0.5		
		**SI (25.2)**	**SI (13.7)**	**SI (11)**	**SI (20.5)**		
Host cell	8-HQ					36.3 ± 2.7	33.6 ± 2.2
	Milt					42.9 ± 1.3	32.8 ± 12.0

## Data Availability

Data is contained within the article.
